# The role of somatostatin in GLP-1-induced inhibition of glucagon secretion in mice

**DOI:** 10.1007/s00125-017-4315-2

**Published:** 2017-05-27

**Authors:** Anne Ørgaard, Jens J. Holst

**Affiliations:** 1 Novo Nordisk Foundation Center for Basic Metabolic Research, Translational Metabolic Physiology, Faculty of Health Sciences, University of Copenhagen, Copenhagen, Denmark; 2 University of Copenhagen, Department of Biomedical Sciences, Faculty of Health Sciences, Blegdamsvej 3B, Bldg 12.2, 2200 Copenhagen N, Denmark

**Keywords:** Antagonist, GLP-1, Glucagon secretion, Hypoglycaemia, Mouse, Pancreas, Paracrine, Perfusion, Somatostatin, SSTR2

## Abstract

**Aims/hypothesis:**

Glucagon-like peptide-1 (GLP-1) receptor agonists are currently used for the treatment of type 2 diabetes. Their main mechanism of action is enhancement of glucose-induced insulin secretion (from increased beta cell glucose sensitivity) and inhibition of glucagon secretion. The latter has been demonstrated to account for about half of their blood glucose-lowering activity. Whereas the effect of GLP-1 on insulin secretion is clearly dependent on ambient glucose concentrations and has been described in detail, the mechanism responsible for the inhibitory effect of GLP-1 on glucagon secretion is heavily debated. Glucagon inhibition is also said to be glucose-dependent, although it is unclear what is meant by this. We hypothesise here that GLP-1 does not inhibit glucagon secretion during hypoglycaemia because the inhibition depends on somatostatin secretion, which in turn is dependent on glucose levels.

**Methods:**

We used the perfused mouse pancreas model to investigate this hypothesis.

**Results:**

We found that, in this model, GLP-1 was able to significantly inhibit glucagon secretion from pancreatic alpha cells at all glucose levels tested: 6.0, 1.5 and 0.5 mmol/l (−27.0%, −37.1%, and −23.6%, respectively), and the decrease in glucagon secretion was invariably accompanied by an increase in somatostatin secretion (+286.8%, +158.7%, and +118.8%, respectively). Specific blockade of somatostatin receptor 2 increased glucagon secretion (+118.8% at 1.5 mmol/l glucose and +162.9% at 6.0 mmol/l glucose) and completely eliminated the inhibitory effect of GLP-1.

**Conclusions/interpretation:**

We have shown here that the glucagon-lowering effect of GLP-1 is entirely mediated through the paracrine actions of somatostatin in the perfused mouse pancreas. However, in this model, the inhibitory effect of GLP-1 was preserved at hypoglycaemic levels, leaving unanswered the question of how this is avoided in vivo in individuals treated with GLP-1 receptor agonists.

## Introduction

Type 2 diabetes is a rapidly increasing health issue worldwide. Over the last decade, glucagon-like peptide-1 (GLP-1) analogues and inhibitors of the GLP-1-degrading enzyme dipeptidyl peptidase-4 (DPP-4) have been implemented as treatments for type 2 diabetes [1, 2]. These drugs stimulate insulin secretion but have the advantage that they carry a low risk of causing hypoglycaemia [1, 3], which is a common side effect of insulin treatment.

The mechanism of action of GLP-1 analogues is to increase the glucose sensitivity of beta cells, thereby enhancing glucose-induced insulin secretion, but they also inhibit glucagon secretion from the alpha cells [4]. This inhibition of glucagon secretion may account for about half of the blood glucose-lowering activity of GLP-1 [5]. Although the effect of GLP-1 on insulin secretion in accordance with the mechanism of action is clearly dependent on ambient glucose concentrations, with considerable amplification at increasing glucose concentrations [6], the glucagon inhibition is also said to be glucose-dependent, although it is unclear what is meant by this. In healthy individuals, glucagon secretion is clearly inhibited by GLP-1 at glucose concentrations around normal fasting levels (4.5–5.5 mmol/l), whereas at higher glucose concentrations, the glucose-induced inhibition predominates and little additional inhibition by GLP-1 is detectable. At lower glucose levels, the inhibitory effect wanes, and at hypoglycaemic levels, which themselves stimulate glucagon secretion, the inhibition by GLP-1 is completely lost [7]. The glucose dependency of both the insulin stimulation and the glucagon inhibition is important clinically, as it means that hypoglycaemia does not normally occur during therapy with GLP-1 analogues.

The mechanism of the inhibitory effect of GLP-1 on glucagon secretion is highly controversial. One possibility is a paracrine inhibition by insulin (the intra-islet hypothesis) [8]. Although this would be compatible with the lack of an inhibitory effect during hypoglycaemia (when insulin secretion is halted), this cannot be the only mechanism, as GLP-1 strongly inhibits glucagon secretion in individuals with long-standing type 1 diabetes [9, 10].

Another possibility is a direct action of GLP-1 on the alpha cells [11]. This has been heavily debated because it is unclear how many alpha cells actually express the GLP-1 receptor (GLP-1R), and if they do, how many GLP-1Rs are actually expressed. Everything from 0% to 100% of pancreatic alpha cells has been reported to express the GLP-1R [12, 13]. One recent study from Rorsman’s group suggested that alpha cells do express GLP-1Rs, but in very low numbers [14]. A major reason for the controversy regarding GLP-1-R localisation has been the lack of specific GLP-1R antibodies [15–17]. Apparently, however, monoclonal antibodies raised against the isolated ectodomain of the GLP-1R show superior specificity. A recent study employing such an antibody raised against the human GLP-1R did not identify any GLP-1Rs on alpha cells in human islets [18], but there might be species differences. We are currently working with a similar antibody against the murine GLP-1R in our own lab in order to further evaluate the localisation of the GLP-1R in the endocrine pancreas.

A third possibility is a paracrine inhibitory action by somatostatin from neighbouring delta cells, as GLP-1 powerfully stimulates somatostatin secretion [19, 20]. Blockade of such an interaction using a somatostatin receptor 2 (SSTR2) antagonist eliminated the inhibitory activity of GLP-1 in perfused rat pancreas [19]. The hypothesis for the current study was that GLP-1 does not inhibit glucagon secretion during hypoglycaemia because the inhibition depends on somatostatin secretion, which in turn—like insulin secretion—is glucose-dependent and almost non-existent at very low glucose concentrations.

## Methods

### Test substances

GLP-1 (7-36-amide), somatostatin-14 and SSTR2 antagonist were ordered from Bachem, Heidelberg, Germany (catalogue nos H-6795, H-1490 and H-6056, respectively). All three were diluted in phosphate buffer containing 1% (wt/vol.) human serum albumin, and stock solutions were stored at −20°C until the experiments were carried out. On the days when the experiments were run, the stocks were diluted in perfusion buffer (see below) and kept at 4°C until infusion. The final perfusate concentrations were 1 nmol/l for GLP-1 and somatostatin, and 100 nmol/l for the SSTR2 antagonist. The positive control, l-arginine, was purchased from Sigma-Aldrich, Munich, Germany (catalogue no. A6969) and dissolved in perfusion buffer immediately prior to infusion (final concentration 10 mmol/l). The final concentrations of GLP-1 in the perfusate were about 10 times higher than the normal physiological peak concentration [21], but similar to those observed after gastric bypass [22], and were chosen in order to minimise variation and compensate for the loss of endogenous factors usually securing full responsiveness in vivo.

### Animals

Female C57BL/6 mice (9–12 weeks, 19–23 g), bred in our own animal facility and with free access to standard rodent chow and water, were used for the experiments. They were housed 2–8 mice per cage under a 12 h light/dark cycle. All animal studies were carried out in accordance with local and international guidelines and with permission from the Danish Veterinary and Food Administration.

### Isolated perfused mouse pancreas

Non-fasted mice were anaesthetised by i.p. injection of ketamine/xylazine (0.1 ml/20 g; ketamine 90 mg/kg [Ketaminol Vet.; MSD Animal Health, Madison, NJ, USA]; xylazine 10 mg/kg [Rompun Vet.; Bayer Animal Health, Leverkusen, Germany]) and the entire abdominal cavity was exposed. The pancreas was then isolated in situ from the circulation as previously described [23].

In brief, the pancreas and duodenum were dissected free of the surrounding tissues, and the abdominal aorta and portal vein were cannulated for single-pass perfusion of the pancreas with a modified Krebs–Ringer bicarbonate buffer containing 0.1% (wt/vol.) BSA, 5% (wt/vol.) dextran T-70 (to provide adequate oncotic pressure; Dextran Products, Scarborough, ON, Canada), 0.5, 1.5 or 6.0 mmol/l glucose, 5 mmol/l each of pyruvate, fumarate and glutamate, and 5 ml/l Vamin (a mixture of essential and non-essential amino acids; Fresenius Kabi, Copenhagen, Denmark) (the last four substrates were omitted in the buffer with 0.5 mmol/l glucose); pH was adjusted to 7.4 with 5 mmol/l HCl. The buffer was oxygenated with a 95% O_2_/5% CO_2_ (vol/vol.) mixture and pre-heated to approximately 39°C.

After establishing the perfusion flow through the pancreas, the mouse was euthanised by perforating the diaphragm. Test substances were infused via a side-arm syringe. The flow rate was kept constant at 2.0 ml/min, and 1 min effluent samples were collected from the venous cannula using a fraction collector (Frac-920; GE Healthcare, Brøndby, Denmark) and stored at −20°C until analysis.

### Hormone analyses

Effluent samples (1 min), collected from the venous cannula, were analysed for glucagon, somatostatin and insulin by in-house RIA. Glucagon concentrations were measured using C-terminally directed assay, employing antiserum 4305, which only detects glucagon of pancreatic origin [24], a technique that has recently been validated against MS [25]. Somatostatin was measured using a rabbit antiserum raised against synthetic cyclic somatostatin (1758), recognising both somatostatin-14 and somatostatin-28 [26, 27]. Insulin concentrations were determined using guinea pig antiserum raised against porcine insulin (2006-3), which cross-reacts strongly with human, rat and mouse insulin [28]. All assays perform well in perfusate with high precision (CV < 5%), sensitivity (<1 pmol/l) and accuracy (recovery of added hormones within ±10% of expected values and linear dilutions).

Hormone concentrations were measured in all 1 min effluent portions, allowing detailed and reliable determination of secretory dynamics. Data were plotted and analysed using GraphPad Prism software, version 6.0 (La Jolla, CA, USA). For statistical analysis of the data, accumulated hormone outputs over 10 min periods of test agent infusions were compared with the preceding 10 min ‘resting periods’. For the l-arginine controls, the maximal peak value was compared with the preceding 10 min ‘resting period’.

## Results

### Responses to GLP-1 at 0.5, 1.5 and 6.0 mmol/l glucose

**Fig. 1:**
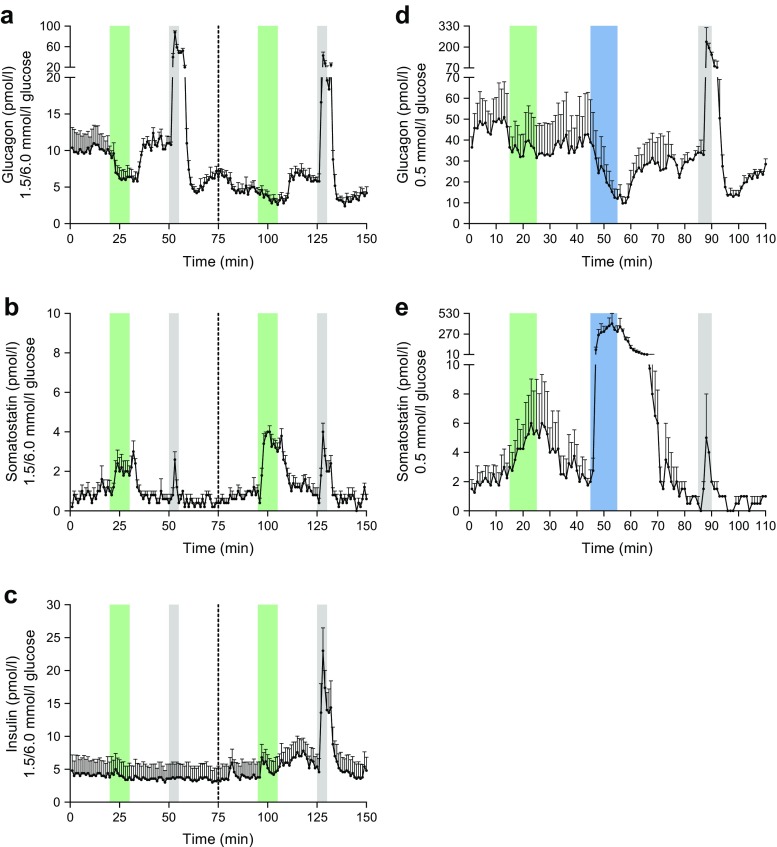
Concentrations of glucagon, somatostatin and insulin in venous effluents from mouse pancreas perfusion experiments with infusion of GLP-1, somatostatin and l-arginine at various glucose concentrations. Green bars, infusion period of 1 nmol/l GLP-1; blue bars, infusion period of 1 nmol/l somatostatin; grey bars, infusion period of 10 mmol/l l-arginine. (**a**–**c**) GLP-1 and l-arginine stimulation at 1.5 and 6.0 mmol/l glucose, *n* = 5. The dotted line at 75 min indicates the mid-experiment transition from 1.5 mmol/l to 6.0 mmol/l glucose in the perfusion medium. (**a**) Glucagon output; (**b**) somatostatin output; (**c**) insulin output. (**d**–**e**) GLP-1, somatostatin and l-arginine stimulation at 0.5 mmol/l glucose (buffer without pyruvate, fumarate, glutamate and Vamin). The first 70 min of data in (**d**–**e**) represents *n* = 4, whereas the end of the protocol (71–110 min), including the positive control, represents only *n* = 2. (**d**) Glucagon output; (**e**) somatostatin output. No insulin was detected at 0.5 mmol/l glucose. l-arginine was used as a positive control. Results are presented as mean + SEM

Initial mouse pancreas perfusion experiments were performed with GLP-1 and l-arginine infusions at 1.5 and 6.0 mmol/l glucose (Fig. 1a–c). The l-arginine was included as a positive control and resulted in large increases in glucagon secretion at both low (1.5 mmol/l) and normal (6.0 mmol/l) glucose levels (+693.9% and +590.3%, respectively) (Fig. 1a,). In parallel to this, l-arginine also elicited strong peaks in somatostatin secretion (+319.4% and 300.0% at 1.5 and 6.0 mmol/l, respectively, i.e. identical fractional increases, but much higher absolute outputs at high glucose; Fig. 1b). At low glucose concentrations (1.5 mmol/l), l-arginine had no effect on insulin secretion, whereas a strong peak in insulin secretion (+274.4%) was seen at 6.0 mmol/l glucose (Fig. 1c). These responses to l-arginine were as expected from previous studies [23, 29].

**Fig. 2:**
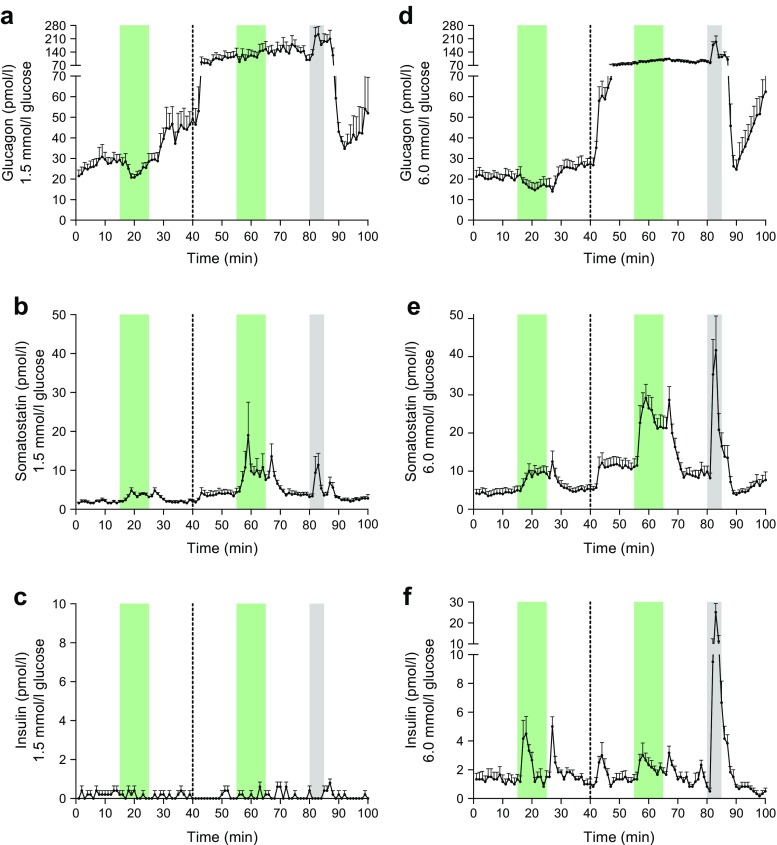
Concentrations of glucagon, somatostatin and insulin in venous effluents from mouse pancreas perfusion experiments with infusion of GLP-1 and SSTR2 antagonist. Green bars, infusion period of 1 nmol/l GLP-1; grey bars, infusion period of 10 mmol/l l-arginine. The dotted line at 40 min indicates the start of the SSTR2 antagonist infusion period, which was continued until completion of the protocol. (**a**–**c**) GLP-1 stimulation without and with the presence of an SSTR2 antagonist at 1.5 mmol/l glucose, *n* = 5. (**a**) Glucagon output; (**b**) somatostatin output; (**c**) insulin output. (**d**–**f**) GLP-1 stimulation without and with the presence of an SSTR2 antagonist at 6 mmol/l glucose, *n* = 6. (**d**) Glucagon output; (**e**) somatostatin output; (**f**) insulin output. l-arginine was used as a positive control. Results are presented as mean + SEM

In our perfusion model, an inhibitory effect of GLP-1 on glucagon secretion (−37.1%) was seen at 1.5 mmol/l glucose—coinciding with an increase in somatostatin secretion (+158.7%) (Fig. 1a, b). There was no change in insulin at this low glucose concentration (Fig. 1c). At 6.0 mmol/l glucose, GLP-1 also decreased glucagon secretion (−27.0%) and concomitantly increased somatostatin secretion (+286.8%) (Fig. 1a, b). GLP-1 also increased insulin secretion (+28.6%) at 6.0 mmol/l glucose (Fig. 1c). The change in glucose concentration from 1.5 to 6.0 mmol/l did not have a major influence on basal somatostatin secretion, which was very low in these experiments, presumably because of the glucose deprivation in the first part of the experiment. However, the absolute responses to both l-arginine and GLP-1 were clearly potentiated at the higher glucose concentrations (see also Fig. 2).

The marked glucagon response to GLP-1 even at 1.5 mmol/l glucose was unexpected, and we therefore studied the effect using an almost substrate-free set-up (buffer without pyruvate, fumarate, glutamate and Vamin) with only 0.5 mmol/l glucose (Fig. 1d, e). The fractional l-arginine responses in this set-up (+673.3 and +350% increases in glucagon and somatostatin secretion, respectively) were comparable to what we observed at 1.5 mmol/l glucose (Fig. 1a, b). In this substrate-deprived buffer, the effect of GLP-1 was preserved, with a significant decrease in glucagon secretion (−23.6%), but paralleled by an increase in somatostatin secretion (+118.8%) (Fig. 1d, e). No insulin was detected.

It should be noted, however, that the pancreas preparations were probably stressed during these substrate-deprived experiments. This was evident from high perfusion pressures and oedema of the pancreatic tissue (data not shown). Out of six attempted experiments, only two were completed to 110 min, two were discontinued 70 min into the protocol, and two were aborted and discarded after 30–40 min. Therefore, the first 70 min of data in Fig. 1d, e represents *n* = 4, whereas the end of the protocol (71–110 min), including the positive control, represents only *n* = 2.

### Responses to GLP-1 in the absence and presence of a SSTR2 antagonist

In the next set of experiments, GLP-1 stimulation was carried out at both low and normal glucose levels in the absence or presence of an SSTR2 antagonist (Fig. 2). The positive control, l-arginine, yielded the expected responses, showing peaks in the secretion of glucagon and somatostatin at both 1.5 and 6.0 mmol/l glucose (+68.2% and +119.7% for glucagon and +206.5% and +365.9% for somatostatin, respectively), but only at 6.0 mmol/l glucose for insulin (+2066.7%) (Fig. 2a–f). At the low glucose levels (1.5 mmol/l), GLP-1 lowered glucagon secretion (−11.5%), and this coincided with an increase in somatostatin secretion (+92.7%).

At 1.5 mmol/l glucose, the addition of the SSTR2 antagonist strongly increased baseline glucagon secretion (+118.8%), and the suppressive effect of GLP-1 on glucagon secretion was eliminated in the presence of the SSTR2 antagonist (Fig. 2a). This was observed even though the somatostatin response to GLP-1 was much higher (+167.5%) during the SSTR2 antagonist infusion period at 1.5 mmol/l glucose (Fig. 2b). At this glucose concentration, basal somatostatin secretion approximately doubled (+101.9%) when the SSTR2 antagonist was applied. Clearly, addition of the SSTR2 antagonist increased the somatostatin responses to both GLP-1 and glucose, presumably by interrupting a negative feedback effect. There was no insulin secretion at 1.5 mmol/l glucose (Fig. 2c).

At normal glucose levels (6 mmol/l; Fig. 2d–f), GLP-1 clearly increased somatostatin secretion (+111.8%) and concomitantly decreased glucagon secretion (−21.7%). When the SSTR2 antagonist was infused, baseline glucagon secretion was dramatically increased (+162.9%), and the inhibitory effect of GLP-1 on glucagon secretion was lost despite a preserved somatostatin response to GLP-1 (+114.2%) (Fig. 2 d, e). As observed with the glucagon response to SSTR2 antagonist infusion, baseline somatostatin secretion also showed a more pronounced increase at 6.0 mmol/l (+121.5%; Fig. 2e) than at 1.5 mmol/l glucose (+101.9%; Fig. 2b) on addition of the SSTR2 antagonist.

GLP-1 infusion triggered a moderate increase in insulin secretion regardless of the presence of the SSTR2 antagonist (+101.7% and +62.3% without or with the SSTR2 antagonist, respectively; Fig. 2f). Addition of the SSTR2 antagonist did not significantly change baseline insulin secretion at 6.0 mmol/l glucose, but did elicit a peak immediately after the infusion was started, much like the typical first-phase response observed for insulin when glucose concentration is increased (Fig. 2f).

## Discussion

Using the perfused mouse pancreas model, we set out to explore why GLP-1-based therapy carries a very low risk of hypoglycaemic events in spite of its ability to inhibit glucagon secretion [1, 3]. We hypothesised that it might be due to a lack of GLP-1-induced somatostatin secretion at low blood glucose levels. GLP-1 stimulation was carried out on perfused mouse pancreas preparations at both low and normal glucose levels, as well as with and without an SSTR2 antagonist. We show here that the basal somatostatin secretion and response to GLP-1 were strongly dependent on perfusate glucose concentrations. Furthermore, the somatostatin response and the inhibition of glucagon secretion were inversely correlated.

In the first series of experiments, we investigated the impact of GLP-1-infusions on the pancreatic secretion of glucagon, somatostatin and insulin. We found that GLP-1 did not affect insulin secretion at low glucose levels (1.5 and 0.5 mmol/l; Fig. 1c, Fig. 2c), whereas GLP-1 increased insulin secretion at normal glucose levels (6.0 mmol/l; Fig. 1c, Fig. 2f). This was consistent with our expectations based on literature describing how the insulinotropic action of GLP-1 depends on simultaneous exposure of the beta cells to glucose [30–33]. However, contrary to what we anticipated, we observed that the inhibitory action of GLP-1 on glucagon secretion was retained at low glucose levels (1.5 and 0.5 mmol/l) (Fig. 1a, d, Fig. 2a). This clearly illustrates the differential actions of GLP-1 on hormone secretion from the alpha and beta cells.

The action of GLP-1 on beta cells is well described and relies on the expression of GLP-1Rs that can be activated and thereby potentiate glucose-induced insulin secretion once plasma glucose reaches a certain threshold level [30, 33]. However, there is much uncertainty regarding the expression of the GLP-1R on alpha cells. Some groups have identified GLP-1Rs on all alpha cells, some have found them on only a subset of alpha cells, and some have been unable to identify them on alpha cells [11, 12, 34–36]. It is therefore highly plausible that the primary route of communication for GLP-1 to the alpha cells is not via direct interaction with these cells, but instead via an indirect route. An indirect mechanism could very well involve somatostatin released from neighbouring delta cells, for which the evidence regarding expression of the GLP-1R is stronger [30, 35, 37].

The results presented in the present study strongly support this hypothesis. According to our original hypothesis, GLP-1 should not be able to inhibit glucagon secretion during hypoglycaemia because no somatostatin would be present under these conditions. In our perfused mouse pancreas model, however, it was not possible to eliminate somatostatin secretion completely by conducting the experiments under even severely hypoglycaemic conditions (Fig. 1b, e, Fig. 2b).

But why is this mechanism lost in humans during hypoglycaemia? One possibility might be that somatostatin tone in the pancreas is usually neurally regulated, as well as being influenced by glucose concentrations [38]. It has previously been shown that somatostatin secretion from the pancreatic delta cells may be under a tonic vagal control in vivo [39]. In our model, all extrinsic neural communication to the richly innervated islets [40] was eliminated owing to the nature of the surgical preparation (see Methods). This may relieve a possible tonic vagal inhibition that could be the cause of the sustained somatostatin secretion seen in our experiments even at extremely low glucose levels. Indeed, efferent vagal activity is greatly stimulated by hypoglycaemia [41, 42]. Nevertheless, our results show that, regardless of glucose concentration, GLP-1-induced inhibition of glucagon secretion is always paralleled by a peak in somatostatin secretion.

In the second series of experiments, we eliminated somatostatin signalling to the alpha cells by applying a specific SSTR2 antagonist. It has previously been shown that SSTR2 is the main somatostatin receptor subtype expressed on rodent alpha cells [43], and it has been suggested that this receptor is responsible for intraislet regulation of glucagon secretion by somatostatin [44–46]. Our results clearly confirm and extend this notion as we observed a dramatic increase in baseline glucagon secretion on addition of the SSTR2 antagonist at both 1.5 and 6.0 mmol/l glucose (Fig. 2a, d); interestingly, glucagon levels were nearly identical, regardless of the glucose concentration, suggesting that the SSTR2 is somehow also involved in the glucose-mediated inhibition of glucagon secretion.

The possibility that somatostatin could be involved in the inhibition of glucagon secretion by glucose is supported by several reports indicating that isolated alpha cells may actually respond to glucose with an increased secretion [36, 47]. The possible role of somatostatin in glucose-mediated inhibition of glucagon secretion will be the subject of further studies. Furthermore, the SSTR2 antagonist also increased baseline somatostatin secretion at both glucose levels (Fig. 2b, e), demonstrating that SSTR2 must be involved in a somatostatin-related autocrine feedback inhibition of the delta cells.

The SSTR2 antagonist did not cause any significant change in baseline insulin secretion (Fig. 2c, f). This is consistent with the fact that somatostatin receptor 5 (SSTR5), rather than SSTR2, may be the main somatostatin receptor on beta cells [45, 48]. The SSTR2 antagonist did, however, elicit a short peak in insulin secretion immediately after addition, which could be caused by a local action of the dramatically increased glucagon secretion at this point [49]. The insulin response to GLP-1 at 6.0 mmol/l glucose was considerably larger before than after addition of the SSTR2 antagonist (+101.7% before vs +62.3% after). Although this difference was not statistically significant, it could probably be ascribed to the greater level of somatostatin present during the second GLP-1 infusion, suppressing insulin secretion through SSTR5 [45, 48].

Most importantly, we show here that GLP-1-induced suppression of glucagon secretion in the mouse pancreas is eliminated in the presence of an SSTR2 antagonist. These results are in line with previous studies on perfused rat pancreas in our own laboratory [19] as well as that of Coy’s group [44]. Other studies identifying somatostatin as a very important paracrine regulator of pancreatic glucagon secretion have been conducted in human islets [50], islets from SSTR2 knockout mice [45] and somatostatin knockout mice both in isolated islets and in vivo [51]. However, Rorsman’s group recently estimated that approximately 1% of the alpha cell population in mouse islets express the GLP-1R, and demonstrated that this small population of GLP-1R-positive cells was sufficient to induce suppression of glucagon secretion in response to GLP-1 over a wide range of glucose concentrations (1–20 mmol/l), based on a theory that limited cAMP responses inhibit glucagon secretion (whereas large responses stimulate secretion). They found no effect of GLP-1 on somatostatin secretion, and addition of a specific SSTR2 antagonist did not influence the inhibitory effect of GLP-1 on glucagon secretion [14].

However, isolated cells are not optimal for studies of intraislet paracrine relationships. In the isolated perfused mouse pancreas, the cytoarchitecture and microvasculature are preserved, thereby ensuring that also paracrine interactions between the islet cells are preserved. In isolated islets, multidirectional diffusion of both extrinsic and intrinsic hormones inevitably occurs during whole-islet incubations, as opposed to perfusion via the natural microcirculation system of the islets.

Insulin has been suggested to play a major role as a paracrine regulator of glucagon secretion (the intraislet hypothesis), but in our experiments at hypoglycaemic glucose levels, insulin (and other beta cell-derived factors, e.g. amylin) is not a significant factor. In rodents, alpha and delta cells are found in close proximity in the periphery of the islets [8, 52, 53], further supporting the existence of paracrine interactions between the alpha and delta cells. Furthermore, the close relationship between alpha and delta cells is also a key point in terms of the potential for translating our results to humans. Thus, regardless of differences in the overall structure and morphology of the islets between rodents and humans, the close relationship between alpha and delta cells appears to be universal [54, 55].

Regarding the possibility that insulin could be involved in suppressing glucagon secretion, we did not, as mentioned, find any evidence of this in our experiments. We found that GLP-1 was able to decrease glucagon secretion and concomitantly increase somatostatin secretion even at very low glucose levels, whereas insulin secretion was unaffected by GLP-1 infusion under these conditions. The sensitivity of the perfusion system with respect to detecting changes in insulin secretion is extreme: the release of even a few femtomoles of insulin would have been detected.

According to the intra-islet hypothesis, insulin acts as a paracrine inhibitor of glucagon secretion [8], but that hypothesis is poorly compatible with the results presented here. First, glucagon is inhibited by GLP-1 even in conditions where insulin is absent (1.5 and 0.5 mmol/l glucose) (Fig. 1a, d, Fig. 2a), and, second, the glucagon response to GLP-1-stimulation is eliminated in the presence of an SSTR2 antagonist despite a preserved GLP-1-induced peak in insulin under these conditions (Fig. 2f). In addition to the present results, the intraislet hypothesis has previously been investigated in our laboratory using the perfused mouse and rat pancreas models and has been found to be inconsistent with the experimental data (unpublished results, B. Svendsen, J. Pedersen, J. de Heer, and J. J. Holst). Furthermore, with regard to the effects of GLP-1, the intraislet hypothesis is also inconsistent with the fact that GLP-1 retains the ability to inhibit glucagon secretion in individuals with type 1 diabetes with no residual beta cell population [9, 10]. A direct effect of GLP-1 on GLP-1Rs expressed on the alpha cells also seems unlikely because a direct GLP-1-induced inhibition of glucagon secretion would not have been eliminated in the presence of an SSTR2 antagonist.

Part of the intra-islet hypothesis rests on a directional flow within the islets from the beta cell core to the mantle, with alpha and delta cells. However, there seems to be agreement in the literature that the alpha and delta cells are generally apposed. Therefore, the paracrine relationship between these cells should not be affected by the intraislet flow, regardless of its direction.

### Conclusions

Our study shows that GLP-1 increases the secretion of somatostatin and concomitantly decreases the secretion of glucagon at all glucose levels. Furthermore, the decreased glucagon secretion in response to GLP-1 infusion is completely eliminated in the presence of an SSTR2 antagonist. Thus, we show here that the glucagon-lowering effect of GLP-1 in mouse pancreas is entirely mediated through the paracrine actions of somatostatin.

## Abbreviations

GLP-1: Glucagon-like peptide-1
GLP-1R: Glucagon-like peptide-1 receptor
SSTR2: Somatostatin receptor 2
SSTR5: Somatostatin receptor 5
